# “In Their Own Words”: A Qualitative Exploration of Lived Experience and Healthcare Professional Perspectives on Evaluating a Digital Intervention for Binge Eating

**DOI:** 10.1002/eat.24539

**Published:** 2025-09-01

**Authors:** Rebecca Murphy, Emma L. Osborne, Nikki Newhouse, John Powell

**Affiliations:** ^1^ Centre for Research on Eating Disorders at Oxford, Department of Psychiatry, Warneford Hospital University of Oxford Oxford UK; ^2^ Nuffield Department of Primary Care Health Sciences University of Oxford Oxford UK

**Keywords:** binge eating disorder, digital intervention, focus groups, healthcare professionals, lived experience, qualitative, randomized controlled trial

## Abstract

**Objective:**

Eating disorders characterized by binge eating are prevalent yet under‐recognized, limiting access to effective care. The digital, programme‐led (self‐help) version of Enhanced Cognitive Behavior Therapy (CBT‐E) offers a potentially scalable treatment. This study gathered insights from individuals with lived experience of binge eating (LE) and healthcare professionals (HCPs) to inform the design of a randomized controlled trial evaluating the intervention's effectiveness and to support early‐stage implementation planning.

**Method:**

Four focus groups were conducted with 20 participants (8 with LE, 12 HCPs). Discussions explored recruitment strategies, participant engagement, meaningful outcome measures, and barriers to implementation. Data were analyzed using thematic analysis.

**Results:**

Two overarching themes were identified: (1) Reach People in Accessible and Supportive Ways, and (2) Be Open to Different Experiences of Progress. Participants emphasized inclusive recruitment and compassionate, hopeful messaging. Stigma and limited recognition of binge eating were cited as recruitment barriers in healthcare settings. Both groups recommended community and online platforms to enhance reach. Participants stressed the importance of outcomes beyond symptom reduction (e.g., emotional well‐being) and qualitative methods to capture recovery narratives. Findings also highlighted implementation‐relevant factors, including how interventions are framed and delivered, and how engagement can be optimized.

**Discussion:**

Perspectives from individuals with LE and HCPs support a person‐centred trial aligned with the needs of those experiencing binge eating and those providing care, while considering both evaluative and implementation priorities. Findings inform strategies to enhance reach and understanding of digital intervention outcomes, contributing to trial designs that are consistent with real‐world care and meaningful to participants.

**Summary**

- Individuals with lived experience of binge eating and healthcare professionals shared their perspectives on the design of a future trial for a digital binge eating intervention.
- Inclusive recruitment strategies and compassionate messaging were viewed as essential to improving access.
- Participants valued outcomes beyond symptom reduction—such as emotional well‐being and progress on a journey—and emphasized the importance of also capturing these “in their own words.”
- Findings support a person‐centered trial design that meets the needs of individuals who experience binge eating and healthcare professionals, promoting real‐world evaluation and practical implementation.

## 1 Introduction

Eating disorders characterized by recurrent binge eating, including bulimia nervosa and binge eating disorder (BED), affect approximately 2 to 5% of the population across their lifetime and are associated with significant psychological and physical health consequences, including depression, anxiety, and cardiovascular and gastrointestinal disorders (Galmiche et al. 2019; Hudson et al. 2007; Kessler et al. 2005; Sheehan and Herman 2015). Binge eating refers to the consumption of large quantities of food while experiencing a loss of control (American Psychiatric Association 2022). Despite their prevalence and burden, these disorders remain under‐recognized and under‐treated across healthcare systems globally (Hilbert 2019; Kazdin et al. 2017). Many individuals are unable to access care due to stigma, limited healthcare provision, high costs, and multiple other barriers (Ali et al. 2017; Becker et al. 2010; Bryant et al. 2022; Liu et al. 2022; Leavey et al. 2011; Penwell et al. 2024).

To address these unmet needs, Enhanced Cognitive Behavior Therapy (CBT‐E; Fairburn 2008) and its associated printed programme‐led (self‐help) version, *Overcoming Binge Eating* (Fairburn 1995, 2013), have been adapted into a digital, programme‐led format, called *Digital CBTe* (https://www.cbte.co/self‐help‐programmes/digital‐cbte/). Guided self‐help (GSH) interventions have demonstrated effectiveness for adults with eating disorders—particularly bulimia nervosa and BED—and are recommended as a first‐line treatment in UK clinical guidelines for adults (Fairburn 2013; Schmidt et al. 2015; Traviss‐Turner et al. 2017; National Institute for Health and Care Excellence 2020). *Digital CBTe* extends this evidence base, delivering structured materials via an app and website, supported by non‐specialist “guides” who encourage engagement and adherence (Baumeister et al. 2014; Linardon et al. 2019). As digital interventions become more common, evaluating them in real‐world contexts is essential for clinical impact and implementation success.

Randomized controlled trials (RCTs) are the gold standard for evaluating treatment efficacy and safety (Schulz et al. 2010; Sackett et al. 1996) but often face major challenges in participant recruitment and retention (Bower et al. 2007; McDonald et al. 2006; Jacques et al. 2022). In a Delphi study of UK Clinical Trials Units (CTUs), research on improving recruitment emerged as the top methodological priority, followed by reducing attrition and selecting appropriate outcomes (Tudur Smith et al. 2014). Directors of UK Clinical Research Collaboration‐registered CTUs were consulted due to their expertise in trial design, delivery, and current methodological challenges. Although rooted in the UK context, these priorities—enhancing recruitment, minimizing attrition, and refining outcome selection—are recognized internationally as enduring challenges in clinical research.

Patient and public involvement (PPI) in trial design is proposed to improve feasibility and relevance (Greenhalgh et al. 2019). The Medical Research Council (MRC) framework for developing and evaluating complex interventions emphasizes early input from those affected by, involved in, or delivering an intervention—such as people with lived experience, healthcare professionals (HCPs), and other contributors (Skivington et al. 2021). Such involvement can address common feasibility challenges: for instance, lived experience and HCP perspectives can inform recruitment strategies to ensure they are acceptable and effective, assess the suitability of outcome measures, and suggest ways to reduce attrition by identifying potential burdens or barriers from user and service viewpoints. Involving people with lived experience and HCPs in trial design may be particularly valuable for BED, where stigma, shame, and under‐recognition create unique barriers to recruitment and engagement. This aligns with the PROMETHEUS programme's recommendation to tailor trial procedures to the specific needs of the target population and condition (Parker et al. 2024).

Another key consideration in trial design is aligning research conditions with how interventions will be delivered in routine practice. Discrepancies—such as highly selective recruitment or researcher‐intensive procedures—can undermine external validity and limit the real‐world applicability of findings (Kennedy‐Martin et al. 2015). This is especially pertinent for digital health interventions, which often receive intensive support in trials that are not feasible at scale (Murray et al. 2016). While such methods may strengthen internal validity, they can compromise generalizability and long‐term implementation.

To address this, the RE‐AIM framework (Reach, Effectiveness, Adoption, Implementation, and Maintenance; Glasgow et al. 1999, 2019) provides a guide for designing and evaluating interventions with population‐level impact in mind. RE‐AIM encourages researchers to consider how an intervention will perform not only under ideal conditions but also when adopted within routine delivery. Applying RE‐AIM during the planning phase helps ensure that trials account for delivery context, scalability, and sustainability—factors essential for bridging the gap between evidence generation and real‐world impact.

In this study, two RE‐AIM domains—*Reach* and *Effectiveness*—are particularly relevant. *Reach* is crucial for BED, where stigma, low awareness, and practical barriers often prevent help‐seeking. Recruitment strategies that mirror real‐life access routes can improve inclusivity and representativeness. *Effectiveness* involves identifying outcomes that matter to those affected by or involved in the intervention. Input from both people with lived experience and HCPs can ensure these outcomes are meaningful from both personal and clinical perspectives. The remaining RE‐AIM domains—Adoption, Implementation, and Maintenance—are more relevant post‐trial but were considered in the development of this intervention to anticipate scale‐up and long‐term delivery challenges.

This study sought insights from people with lived experience of binge eating—referred to here as lived experience contributors (LECs)—and HCPs in specialist eating disorder services to inform the design of a future RCT evaluating a digital GSH programme for binge eating. The trial will mirror real‐world implementation through recruitment strategies, delivery models, and outcome assessments aligned with routine use. To support this, qualitative focus groups with LECs and HCPs explored recruitment pathways, outcome priorities, and the acceptability of trial procedures. This formative work aims to lay the foundation for trials with strong potential for real‐world impact.

## 2 Method

### 2.1 Study Design and Setting

This qualitative study used focus groups to explore LEC and HCP perspectives on designing and implementing an RCT for a digital GSH programme for binge eating. Focus groups were chosen to encourage rich, interactive discussion and allow participants to compare and elaborate on their experiences and views. The study is reported in accordance with the Consolidated Criteria for Reporting Qualitative Research (COREQ) checklist (Tong et al. 2007; see Supporting Information).

### 2.2 Recruitment

Four focus groups were planned—two with LECs and two with HCPs—each with 4–6 participants to enable meaningful dialogue while ensuring all voices were heard. Sample size was guided by ‘information power’ (Malterud et al. 2016), which suggests that studies with a clearly defined aim and relevant participants may require fewer individuals to generate meaningful insights. Participants were recruited via the Centre for Research on Eating Disorders at Oxford (CREDO) contributor network, using purposive sampling to capture variation in age, gender, ethnicity, and professional or experiential background. While some demographic diversity was achieved, scheduling constraints limited full heterogeneity.

Some participants had dual perspectives (e.g., personal and professional) and chose the group they most identified with. Recruitment materials were distributed via targeted email invitations based on demographic and role data previously collected by CREDO. Eligibility was screened via Qualtrics, and informed consent obtained electronically. Inclusion criteria were: (1) current or past residence in the UK or Ireland; (2) age ≥ 18; and (3) lived experience of binge eating, or professional experience supporting individuals with binge eating.

### 2.3 Procedure

Focus groups were held via Microsoft Teams and lasted 60–90 min. A brief introductory presentation outlined the digital GSH programme and the basic RCT structure to ensure a shared understanding. The topic guide, developed with input from a separate PPI group with lived experience of binge eating (not study participants) and reviewed for clarity, covered recruitment strategies, participant engagement, and outcome measurement. Sessions were scheduled flexibly, including outside typical working hours, to maximize accessibility. Each participant received a £15 Amazon e‐voucher. Groups were facilitated by R.M. with support from E.L.O., who also took field notes and promoted inclusive discussion. Only participants and researchers were present, and each group met once.

### 2.4 Data Collection and Analysis

All sessions were video recorded, transcribed verbatim, and anonymised by removing names. Only first or preferred names were used during groups, and all quotes were checked to ensure they could not be linked to individuals. Procedures followed the approved ethics protocol.

Data were analyzed using Braun and Clarke's (2006) thematic analysis, following an iterative process of familiarization, coding, and theme development. A codebook approach was chosen for its flexibility in capturing both shared and divergent perspectives, with a focus on trial design. Guided by a constructivist paradigm, this approach recognized the co‐construction of meaning between researchers and participants. Analysis was conducted collaboratively by R.M. and E.L.O., with input from N.N. and J.P. to enhance rigor. A coding tree was not used, as interpretive theme development was prioritized over hierarchical structuring.

Transcripts were organized and analyzed manually in Microsoft Excel to support in‐depth engagement with the data. Participants were not asked to review transcripts or findings, minimizing burden and reflecting the constructivist view that knowledge is co‐produced rather than verified post hoc.

### 2.5 Researcher Characteristics and Reflexivity

The multidisciplinary team brought varied expertise: R.M. (female, DClinPsy), a clinical psychologist with over 20 years' experience in eating disorders, expertise in CBT‐E, and a central role in developing *Digital CBTe*; E.L.O. (female, MRes, MA), an eating disorders researcher ensuring methodological rigor and a broader perspective; N.N. (female, PhD), a qualitative methods specialist with expertise in human–computer interaction in health and social care; and J.P. (male, MB, PhD), a digital health researcher and public health physician focused on feasibility and implementation. PPI contributors of mixed genders and lived experience informed the design and delivery of the focus groups, advising on recruitment, informed consent, topic guide content, theme wording, and accessibility (e.g., online delivery, flexible scheduling).

Reflexivity was embedded throughout. The team regularly discussed how their backgrounds might have influenced data collection, analysis, and interpretation. Both facilitators had qualitative methods training and experience with the target population; some participants had prior familiarity with R.M. and E.L.O., and a few knew the digital GSH programme from previous studies. These relationships were acknowledged and reflected upon during data interpretation.

Attention to power dynamics drew on Hopkins et al. (2024) and co‐production principles. All participants were compensated for their time, with payment framed as recognition of meaningful work. Focus group facilitators encouraged diverse perspectives, amplified quieter voices, and avoided jargon or defined it when necessary. PPI contributors provided feedback on theme wording to ensure shared language. Multiple identities (e.g., LEC and HCP, or carer) were acknowledged from the outset, and facilitators monitored emotional tone to maintain a psychologically safe environment.

## 3 Results

### 3.1 Participant Characteristics

Twenty participants took part: eight LECs and 12 HCPs. All LECs self‐identified as having lived experience of recurrent binge eating, most with long‐standing eating difficulties (six > 20 years) and prior psychological treatment, which included dietary advice, medication, or hospitalization. All HCPs had current or past experience of working in eating disorder services, representing roles such as psychologists, therapists, psychiatrists, and service managers.

Some participants held dual perspectives: one LEC also supported family/friends with experience of binge eating; two HCPs also had lived experience of binge eating, one of whom also supported family/friends. No participants withdrew after consent.

The mean age was 52.9 years for LECs (range 36–68) and 42.6 years for HCPs (range 26–62), with an overall sample mean of 46.7. Fourteen participants identified as women and six as men; 15 identified as White British or White Irish, and five as other White or mixed ethnic backgrounds. Three LECs had previously participated in a study of the digital programme.

### 3.2 Overview of Themes

Participants emphasized the importance of a trial design that reflects the real‐world experiences of people with BED, who often face stigma, under‐recognition in healthcare, and limited access to support. Recruitment solely through healthcare was seen as inadequate; participants recommended using digital platforms and community outreach, paired with compassionate, non‐weight‐focused messaging.

They also called for a broader definition of treatment success. While symptom reduction was valued, outcomes such as emotional wellbeing, understanding of the eating problem, and daily functioning were also considered important. Participants also advocated for qualitative assessments to capture change in their own words. Recovery was viewed as an ongoing journey, with progress valued at all stages.

Two overarching themes were identified:

1. Reach People in Accessible and Supportive Ways
2. Be Open to Different Experiences of Progress

**FIGURE 1:**
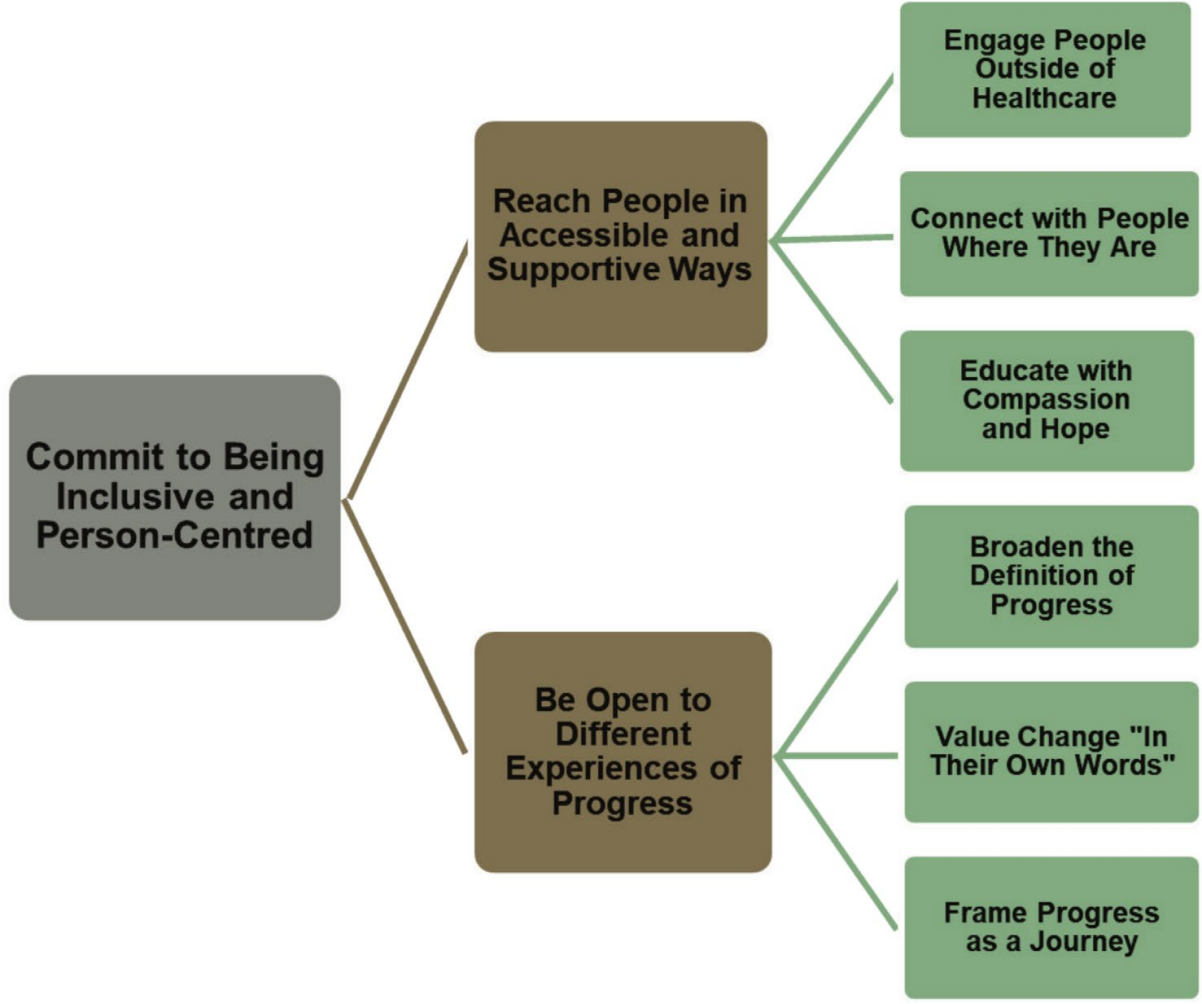
Thematic map of lived experience and healthcare professional perspectives on trial design for digital binge eating treatments.

Each theme includes sub‐themes reflecting priorities for an inclusive, person‐centered evaluation. Illustrative quotes are embedded in the text, and Figure 1 presents the thematic map.

## 4 Themes

### 4.1 Reach People in Accessible and Supportive Ways

Participants agreed that traditional recruitment through healthcare settings would likely exclude many individuals who experience binge eating, citing multiple barriers that would reduce the likelihood that people who experience binge eating would present for treatment in conventional services. They offered practical alternatives and guidance to address these challenges.

#### 4.1.1 Engage People Outside of Healthcare

While some HCPs initially viewed clinical environments such as general practitioner (GP) practices or eating disorder services as logical recruitment settings, LECs disagreed. Both groups, however, highlighted barriers to recruitment in these environments, noting that shame often discourages help‐seeking. As one HCP observed, “this particular client group is unlikely to come forward or… present at services… they're on very long wait lists… and that sense of shame that's associated with it” [HCP 7]. Additional challenges included lack of problem recognition and understanding by GPs and long waits. One LEC explained, “In my area it can take up to six months to see a GP, so scrap surgeries—we're not even allowed into the surgery unless we have an appointment” [LEC 6]. Others added, “GPs haven't got time” [LEC 2] to discuss research. [In the UK, the term “surgery” refers to a GP's office or clinic, rather than to a surgical procedure.]

Several participants felt binge eating is often overlooked or misunderstood in primary care. LECs, in particular, described feeling dismissed or reduced to their weight status. One stated, “the NHS is… geared towards… weight loss” [LEC 8]. Both HCPs and LECs described BED as receiving less attention and care than other diagnoses, leading one HCP to refer to it as the “poor relative” in eating disorders.

#### 4.1.2 Connect With People Where They Are

To address these barriers, participants recommended reaching people in the settings they naturally turn to for support—both online and in local communities. While this view was broadly shared, LECs and HCPs differed in how and why they proposed it. LECs emphasized the self‐directed nature of support‐seeking, often viewing binge eating as a personal struggle rather than a clinical condition. They described seeking information and connection through social media, forums, and health‐related websites. One LEC explained, “I go digging round on the internet… all the groups where I think there might be a shared interest” [LEC 3]. Another noted, “we all work quite chaotic hours and don't necessarily, you know, go to a doctors' surgery, we try and find a solution ourselves” [LEC 3].

HCPs framed community‐based recruitment more as a strategic outreach method to engage people unlikely to present in clinical settings: “going to the areas where there might be a lot of people with these problems” [HCP 3]. LECs also offered targeted suggestions for underserved groups, such as “The way to reach men is through sports. Any kind of sports channels” [LEC 8].

#### 4.1.3 Educate With Compassion and Hope

In addition to identifying *where* to recruit, participants stressed the importance of *how* recruitment materials should be framed. They advocated for compassionate, hopeful messaging that recognizes BED as a real, treatable disorder—not a personal failing or simple overeating.

HCPs tended to focus on education to raise awareness and increase help‐seeking: “There is so much shame associated with binge eating… some people don't even consider it is an eating disorder at all” [HCP 7], and using relatable questions like, “do you sometimes overeat and you can't stop?” [HCP 12]. LECs drew on personal experiences of invalidation, describing how help‐seeking was often dismissed or redirected towards weight: “That just shuts you down… you've tried that for years” [LEC 1]; “I really resented being spoken down to… as if I didn't understand that I should eat less. But the problem was the binge eating” [LEC 8]. This underscored the need to focus messaging on eating behaviors rather than weight.

Across groups, participants agreed that validating language is key to reducing shame and encouraging engagement. BED was seen as lacking visibility and “isn't really taken seriously” [LEC 1]. As one HCP explained, “It's really important to instil hope… a lot of clients have a huge shame and history of failed attempts” [HCP 1]. Messaging should reassure potential participants that, while the digital format may be new, the treatment is evidence‐based. However, they cautioned against unrealistic promises and endorsed framing that offers realistic hope.

### 4.2 Be Open to Different Experiences of Progress

Participants called for broader, more personalized measures of success in BED treatment. While symptom reduction was important, emotional wellbeing, improved understanding, and meaningful life changes were considered equally vital. Recovery was described as a journey rather than a fixed endpoint, and participants recommended outcome measures that reflect this diversity, including qualitative assessments to capture individual experiences.

#### 4.2.1 Broaden the Definition of Progress

While reducing binge eating episodes was considered important, participants also highlighted other recovery indicators, such as self‐compassion around eating behaviors, improved strategies for managing emotions and triggers, and positive life impacts. “It's how people are managing their emotions… not using food as comfort” [HCP 6]. Another noted, “For most people, it's the impact [binge eating] has on the rest of their life that stands out” [HCP 3].

Views varied on which outcomes mattered most. Some prioritized an immediate symptom reduction, while others highlighted different measures of success. For example, one LEC emphasized longer‐term resilience: “can you maintain… recovery… when you're put under stress. That's the real question” [LEC 1].

#### 4.2.2 Value Change “In Their Own Words”

Many LECs stressed the importance of recognizing self‐defined progress and using qualitative measures to capture it. “Sometimes with the questionnaires… you're like trying to fit into a box… you don't fit any of the answers” [LEC 1]. They valued “an opportunity to actually express the changes…in your own words” [LEC 1], with one suggesting “a one‐to‐one interview… to get to …underlying issues and…find out how people themselves have benefitted… In their own words.” [LEC 8].

HCPs similarly supported open‐ended questions to capture the broader impacts, such as, “has any area of your life been changed as a result of this?” [HCP 5]. They noted it would be valuable to hear about improvements in “their family life, their work life, social, you know, they can go to work now or they go out to dinner or something like that or, you know, marital relationship” [HCP 5].

#### 4.2.3 Frame Progress as a Journey

Both HCPs and LECs described progress as a journey, where each step is valued and can contribute to ongoing recovery. This might include improved understanding of the eating disorder and reduced shame, even without full recovery. One LEC reflected, “The last thing to change was my behaviour… the early changes were in how I spoke to myself, how I approached food and my body… that's really difficult… but that had to come first” [LEC 1].

Digital GSH was seen as a valuable starting point, particularly where other services are limited: “It may be all some people need… for others, a pathway to something else” [LEC 8]. One HCP similarly described “Setting them off on the right path… giving them the skills… and toolbox… to get going on their recovery journey” [HCP 9].

## 5 Discussion

This study gathered lived experience and healthcare professional perspectives to inform the design of an RCT evaluating a digital GSH intervention for binge eating. Participants identified two priorities for optimizing trial design: inclusive recruitment strategies and a broader, person‐centred approach to outcome evaluation. These insights support trial feasibility and relevance while contributing to early‐stage implementation planning—essential for future scalability and sustainability.

To our knowledge, this is one of the first studies to integrate both perspectives into early‐stage trial design using the RE‐AIM framework. By aligning feedback with the Reach and Effectiveness domains, our application of RE‐AIM extended beyond post‐implementation evaluation to guide the practical and person‐centered design of clinical trials from the outset.

### 5.1 Implications for Recruitment and Reach

Participants emphasized the need to broaden recruitment efforts beyond healthcare settings, noting that many people who experience binge eating do not access clinical services due to stigma, under‐recognition, and long wait times. These barriers align with research on stigma (Aird et al. 2025; Kurdak et al. 2023; Salvia et al. 2022), trivialization of BED (Nwuba 2023), limited primary care knowledge (Field et al. 2024; Johns et al. 2019), and low referral rates (Phillipou et al. 2023).

To improve reach, participants recommended strategies reflecting real‐world behaviors, including digital platforms, community organizations, and informal networks. Advertising in familiar online and offline settings was seen as critical for accessibility and stigma reduction, consistent with calls for community‐based recruitment to engage underrepresented groups, such as men and ethnic minorities (Carrino et al. 2023), and an expert consensus advocating multiple access routes beyond traditional services, including the internet and community settings (Davey et al. 2023).

Participants also noted that many perceive binge eating as a personal struggle rather than a clinical condition, supporting the use of educational messaging that frames BED as a recognized, treatable disorder. This echoes Radunz et al. (2023), who identified “denial of illness”—often from individuals not recognizing their symptoms as indicative of a disorder requiring professional support—and doubts about treatment availability as barriers to help‐seeking. Emphasizing the intervention's evidence base, even if with a new mode of delivery, was seen as important for building hope and trust.

While there was notable convergence, distinct emphases emerged: LECs were more likely to describe emotional invalidation in healthcare and the importance of feeling understood, while HCPs highlighted systemic delivery challenges such as limited training and service constraints. This echoes earlier work on divergent perspectives in eating disorder care (e.g., de la Rie et al. 2007) and supports the involvement of both groups to strengthen the inclusivity and contextual grounding of trial design.

These findings align with the RE‐AIM framework's *Reach* domain, which prioritizes engaging the intended population. Community‐based, inclusive, and education‐focused strategies can enhance reach, external validity, and scale‐up potential. Although digital interventions offer promise for reaching underserved individuals (Linardon et al. 2019), their impact depends on where, how, and with what messaging people are engaged. Our findings extend prior work by not only reaffirming known barriers to care (e.g., stigma and lack of recognition; Ali et al. 2017) but by identifying LEC‐ and HCP‐driven strategies for mitigating these barriers.

For example, while food pantry‐based outreach has recently been explored for intervention dissemination (Graham et al. 2024) and community recruitment has been shown to improve representativeness in eating disorder research (Carrino et al. 2023), our study contributes novel insights on *how* to frame recruitment materials to enhance engagement. Participants recommended messaging that emphasizes hope, acknowledges the emotional distress associated with binge eating, and avoids weight‐centric language—operationalizing broader calls for accessible care pathways. Emotional safety (i.e., not feeling judged, dismissed, or shamed) was seen as critical, with several noting that language in recruitment materials could either reinforce or reduce shame. One LEC reported that weight‐focused advice “just shuts you down”, while another “really resented being spoken down to”. Practical suggestions included reaching men through sports networks and using online spaces where individuals self‐identify with binge eating behaviors, even without a formal diagnosis—approaches not widely explored in prior literature.

These recommendations also point to broader systemic needs, including enhanced HCP training, increasing awareness of BED, and more trained therapists. While UK initiatives are underway (e.g., Novogrudsky et al. 2024), further expansion is needed (Ayton and Ibrahim 2018). LEC concerns about healthcare barriers highlight the value of access routes without clinical gatekeeping—such as community outreach and self‐referral—to improve equity. Consistent with this, expert consensus supports non‐clinical pathways that bypass primary care to widen access to programme‐led interventions (Davey et al. 2023).

### 5.2 Implications for Measuring Treatment Progress

Participants advocated for a broader, person‐centered approach to outcome assessment. While symptom reduction was valued, other outcomes—such as emotional wellbeing, understanding of the eating problem, and daily functioning—were also seen as important, aligning with emerging consensus that recovery should be defined by multidimensional outcomes rather than symptom remission alone (Austin et al. 2023; Hanegraaf et al. 2024). This perspective reflects the RE‐AIM framework's *Effectiveness* domain, which emphasizes meaningful, patient‐centered outcomes beyond symptom change.

Participants also supported qualitative assessments to capture individual experiences of progress, consistent with the MRC framework's focus on contributor perspectives (Skivington et al. 2021) and the growing use of mixed‐methods designs in implementation research. Participants stressed that digital interventions should be situated as part of a broader recovery journey—sufficient for some, but a stepping‐stone to further treatment for others. Framing recovery in this way may help manage expectations for both patients and healthcare providers, and support engagement and adherence, which are essential for successful implementation.

### 5.3 Limitations and Future Research

This study has several limitations. Conducting focus groups online may have introduced selection bias by excluding those less comfortable with digital communication—though this format was endorsed by our PPI contributors and reflected the digital nature of the intervention. As with all group methods, discussion dynamics may have influenced whose voices were heard. Although the topic guide included participant engagement, no specific insights were shared on retention strategies—an important area for future research, particularly in digital intervention trials. Participant characteristics also warrant consideration: the sample had the digital literacy, confidence, and time to engage with research, meaning discussions about improving reach were shaped by those already reachable. While efforts were made to include participants from varied genders and ethnic groups, most participants were women and White. The study focused on a digital GSH intervention for binge eating, and so some perspectives—such as those relating to stigma—may be specific to this context. Moreover, although digital GSH shows promise for improving access, it is not a panacea. Barriers such as unstable digital access, complex support needs, financial constraints, and low self‐efficacy (Asaria 2025; Choy et al. 2024) underscore the need for flexible, blended, or stepped‐care models and the importance of embedding equity considerations from the outset.

Insights from this study will inform a forthcoming feasibility RCT of Supported (guided) *Digital CBTe*, ensuring an implementation‐informed, person‐centered evaluation. Grounding intervention and evaluation design in the perspectives of those directly affected can strengthen both relevance and translational impact (Boaz et al. 2018; Hudson et al. 2020). Further research using frameworks such as RE‐AIM and NASSS (nonadoption, abandonment, scale‐up, spread, and sustainability; Greenhalgh et al. 2017) is needed to assess other implementation outcomes, including feasibility, adoption, fidelity, and sustainability (Proctor et al. 2011).

## 6 Conclusion

This study explored LEC and HCP perspectives to inform the design of a future RCT for a digital GSH programme for binge eating. Participants prioritized inclusive, community‐based recruitment, compassionate and non‐weight‐focused messaging, and broader, person‐centred outcome measures. Integrating these insights—which address common barriers and reflect real‐world contexts—into trial design can enhance feasibility, relevance, and real‐world applicability, supporting both immediate trial success and long‐term scalability.

## Author Contributions

**Rebecca Murphy:** conceptualization, methodology, investigation, formal analysis, writing – original draft, writing – review and editing, project administration, funding acquisition. **Emma L. Osborne:** conceptualization, methodology, investigation, formal analysis, writing – original draft, writing – review and editing. **Nikki Newhouse:** conceptualization, methodology, writing – review and editing, supervision. **John Powell:** conceptualization, methodology, funding acquisition, writing – review and editing, supervision.

## Ethics Statement

This study was reviewed and approved by the University of Oxford Central University Research Ethics Committee (CUREC) [Reference: R70660/RE001]. All participants provided written informed consent prior to participation, and the study was conducted in accordance with the Declaration of Helsinki.

## Conflicts of Interest

R.M. is a founder, shareholder, and consultant for Credo Therapies. E.L.O. is a part‐time employee of Credo Therapies. J.P. is an advisor for Credo Therapies. Credo Therapies is an impact‐driven company that has an exclusive license to a digital programme‐led version of Enhanced Cognitive Behavior Therapy.

## Supporting information


**Data S1:** Supporting information.

## Data Availability

Research data are not shared.
