# Supplementary material for: “In Their Own Words”: A Qualitative Exploration of Lived Experience and Healthcare Professional Perspectives on Evaluating a Digital Intervention for Binge Eating
Source: Int J Eat Disord. 2025 Sep 1;58(12):2271–80. doi: 10.1002/eat.24539 (PMC12703220; doi:10.1002/eat.24539)
Supplement: Supplementary file 1 — Data S1: Supporting information. [file EAT-58-2271-s001.pdf]

Consolidated criteria for reporting qualitative studies (COREQ): 32-item checklist

Please indicate in which section each item has been reported in your manuscript. If you do not feel an item applies to your manuscript, please enter N/A.

For further information about the COREQ guidelines, please see Tong *et al.*, 2017: https://doi.org/10.1093/intqhc/mzm042

No.	Item	Description	Section #
**Domain 1: Research team and reflexivity**			
Personal characteristics			
1.	Interviewer/facilitator	Which author/s conducted the interview or focus group?	Methods: Researcher Characteristics and Reflexivity
2.	Credentials	What were the researcher's credentials? *E.g. PhD, MD*	Methods: Researcher Characteristics and Reflexivity
3.	Occupation	What was their occupation at the time of the study?	Methods: Researcher Characteristics and Reflexivity
4.	Gender	Was the researcher male or female?	Methods: Researcher Characteristics and Reflexivity
5.	Experience and training	What experience or training did the researcher have?	Methods: Researcher Characteristics and Reflexivity
Relationship with participants			
6.	Relationship established	Was a relationship established prior to study commencement?	Methods: Researcher Characteristics and Reflexivity
7.	Participant knowledge of the interviewer	What did the participants know about the researcher? *E.g. Personal goals, reasons for doing the research*	Methods: Researcher Characteristics and Reflexivity
8.	Interviewer characteristics	What characteristics were reported about the interviewer/facilitator? *E.g. Bias, assumptions, reasons and interests in the research topic*	Methods: Researcher Characteristics and Reflexivity
**Domain 2: Study design**			
Theoretical framework			
9.	Methodological orientation and theory	What methodological orientation was stated to underpin the study? *E.g. grounded theory, discourse analysis, ethnography, phenomenology, content analysis*	Methods: Data Collection and Analysis
Participant selection			
10.	Sampling	How were participants selected? *E.g. purposive, convenience, consecutive, snowball*	Methods: Recruitment
11.	Method of approach	How were participants approached? *E.g. face-to-face, telephone, mail, email*	Methods: Recruitment
12.	Sample size	How many participants were in the study?	Results: Participant Characteristics
13.	Non-participation	How many people refused to participate or dropped out? What were the reasons for this?	Results: Participant Characteristics
Setting			
14.	Setting of data collection	Where was the data collected? *E.g. home, clinic, workplace*	Methods: Procedure
15.	Presence of non-participants	Was anyone else present besides the participants and researchers?	Methods: Procedure

16.	Description of sample	What are the important characteristics of the sample? *E.g. demographic data, date*	Results: Participant Characteristics
Data collection			
17.	Interview guide	Were questions, prompts, guides provided by the authors? Was it pilot tested?	Methods: Procedure
18.	Repeat interviews	Were repeat interviews carried out? If yes, how many?	Methods: Procedure
19.	Audio/visual recording	Did the research use audio or visual recording to collect the data?	Methods: Procedure
20.	Field notes	Were field notes made during and/or after the interview or focus group?	Methods: Data Collection and Analysis
21.	Duration	What was the duration of the interviews or focus group?	Methods: Procedure
22.	Data saturation	Was data saturation discussed?	Methods: Recruitment
23.	Transcripts returned	Were transcripts returned to participants for comment and/or correction?	Methods: Data Collection and Analysis
**Domain 3: analysis and findings**			
Data analysis			
24.	Number of data coders	How many data coders coded the data?	Methods: Data Collection and Analysis
25.	Description of the coding tree	Did authors provide a description of the coding tree?	Methods: Data Collection and Analysis
26.	Derivation of themes	Were themes identified in advance or derived from the data?	Methods: Data Collection and Analysis
27.	Software	What software, if applicable, was used to manage the data?	Methods: Data Collection and Analysis
28.	Participant checking	Did participants provide feedback on the findings?	Methods: Data Collection and Analysis
Reporting			
29.	Quotations presented	Were participant quotations presented to illustrate the themes / findings? Was each quotation identified? *E.g. Participant number*	Results: Overview of Themes<br>Results: Themes
30.	Data and findings consistent	Was there consistency between the data presented and the findings?	Results: Overview of Themes<br>Results: Themes
31.	Clarity of major themes	Were major themes clearly presented in the findings?	Results: Overview of Themes<br>Results: Themes
32.	Clarity of minor themes	Is there a description of diverse cases or discussion of minor themes?	Results: Overview of Themes<br>Results: Themes

When submitting your manuscript via the online submission form, please upload the completed checklist as a Figure/supplementary file.

If you would like this checklist to be included alongside your article, we ask that you upload the completed checklist to an online repository and include the guideline type, name of the repository, DOI and license in the *Data availability* section of your manuscript.

Developed from: Allison Tong, Peter Sainsbury, Jonathan Craig, Consolidated criteria for reporting qualitative research (COREQ): a 32-item checklist for interviews and focus groups, International Journal for Quality in Health Care, Volume 19, Issue 6, December 2007, Pages 349–357, https://doi.org/10.1093/intqhc/mzm042
